# Prediction of lung cancer risk in a Chinese population using a multifactorial genetic model

**DOI:** 10.1186/1471-2350-13-118

**Published:** 2012-12-10

**Authors:** Huan Li, Lixin Yang, Xueying Zhao, Jiucun Wang, Ji Qian, Hongyan Chen, Weiwei Fan, Hongcheng Liu, Li Jin, Weimin Wang, Daru Lu

**Affiliations:** 1 State Key Laboratory of Genetic Engineering and MOE Key Laboratory of Contemporary Anthropology, Institute of Genetics, School of Life Sciences, Fudan University, Handan Rd, Shanghai, 200433, China; 2 Department of Cardiothoracic Surgery, Changhai Hospital of Shanghai, Second Military Medical University, Shanghai, China; 3 Zhejiang Provincial Key Laboratory of Biometrology and Inspection and Quarantine Technique, College of Life Sciences, China Jiliang University, Hangzhou, China; 4 Zhejiang Provincial Key Laboratory of Biometrology and Inspection & Quarantine, China Jiliang University, Hangzhou, 310018, China

**Keywords:** Chinese, Cumulative risk, Genetic risk score, Lung cancer, Risk assessment

## Abstract

**Background:**

Lung cancer is a complex polygenic disease. Although recent genome-wide association (GWA) studies have identified multiple susceptibility loci for lung cancer, most of these variants have not been validated in a Chinese population. In this study, we investigated whether a genetic risk score combining multiple.

**Methods:**

Five single-nucleotide polymorphisms (SNPs) identified in previous GWA or large cohort studies were genotyped in 5068 Chinese case–control subjects. The genetic risk score (GRS) based on these SNPs was estimated by two approaches: a simple risk alleles count (cGRS) and a weighted (wGRS) method. The area under the receiver operating characteristic (ROC) curve (AUC) in combination with the bootstrap resampling method was used to assess the predictive performance of the genetic risk score for lung cancer.

**Results:**

Four independent SNPs (rs2736100, rs402710, rs4488809 and rs4083914), were found to be associated with a risk of lung cancer. The wGRS based on these four SNPs was a better predictor than cGRS. Using a liability threshold model, we estimated that these four SNPs accounted for only 4.02% of genetic variance in lung cancer. Smoking history contributed significantly to lung cancer (*P* < 0.001) risk [AUC = 0.619 (0.603-0.634)], and incorporated with wGRS gave an AUC value of 0.639 (0.621-0.652) after adjustment for over-fitting. This model shows promise for assessing lung cancer risk in a Chinese population.

**Conclusion:**

Our results indicate that although genetic variants related to lung cancer only added moderate discriminatory accuracy, it still improved the predictive ability of the assessment model in Chinese population.

## Background

Lung cancer is one of the leading causes of cancer death worldwide [1,2]. Most patients are diagnosed at an advanced stage, so are not able to undergo surgical removal of tumors [1]. As a result, the overall 5-year survival rate is low. Early stage detection when treatment might be more effective, would therefore help reduce lung cancer mortality. For this reason, a well-established assessment model that could identify individuals at high risk would greatly benefit patients, clinicians and researchers.

Lung cancer is a polygenic disease, for which many genetic factors appear to play an important role in disease development [2,3]. During the past three years, several genome-wide association (GWA) studies have identified a number of genetic susceptibility loci associated with lung cancer risk [4-9], but most of these studies were conducted in populations of European descent, and many identified risk alleles have not been adequately evaluated in Asian populations.

In addition, when examined individually, each of the genetic susceptibility loci only confers a small to moderate disease risk, and is of limited utility in risk prediction. It is possible that combining multiple disease-related loci with modest effects into a genetic risk score (GRS) may be useful to identify subgroups that are at high risk of lung cancer [10,11]. Several lung cancer risk assessment models have been proposed, including the Bach model, Spize model, and Liverpool Lung Project (LLP) model [12-15]. However, most predictors from these models focus on demographic and clinical factors, and, to our knowledge, no report has quantified the risk of lung cancer using a combination of newly identified risk loci in a Chinese population.

In this case–control study, we evaluate the discriminatory and predictive ability of the cumulative effect of several SNPs associated with lung cancer risk in populations of European descent, and estimate the proportion of genetic variants explained by the selected risk loci in a Chinese population.

## Methods

### Subjects

A total of 2,283 lung cancer cases and 2,785 cancer-free controls (from Shanghai Zhongshan Hospital, Shanghai Chest Hospital, First Affiliated Hospital of Nanjing Medical University, Beijing Union Medical College Hospital, and Wuhan Union Hospital, China) who were genetically unrelated Han Chinese were enrolled in this study. Eligible patients had histopathologically confirmed lung cancer, and with no previous cancer history and were no receiving radiotherapy or chemotherapy for other condition. Control participants were randomly selected from individuals receiving routine physical examinations in local hospitals or those who participated in a community-based screening program of non-communicable diseases. They were frequency-matched to the cases according to age, gender and residential area.

Information on smoking was collected by means of interviews. Individuals who had smoked less than one cigarette per day for less than one year of their lifetime, or less, were defined as nonsmokers. The remaining individuals were divided into light and heavy smokers according to the threshold of 25 pack years (median pack years in the controls). All participants provided written informed consent for study participation with approval from institutional review boards of each participating institution.

### Selection of genetic risk factors and genotyping

We reviewed the literature on GWAS and large cohort studies published up until June, 2011, and selected those lung cancer risk SNPs from GWAS demonstrating *p* < 5E-6 or from large cohort studies with evidence of replication at *p* < 0.05. In total, Five SNPs were selected for analysis (Table 1).

**Table 1:** Selected SNPs associated with lung cancer^*^

**First author, year (reference)**	**SNP**	**Gene**	**Chromosomal location**	**Genotyping rate**	**Risk allele**	**Reported OR (95% CI) ‡**
McKay, 2008 [4]	rs2736100	TERT	5p15	99.5%	C	1.18 (1.10-1.26)
McKay, 2008 [4]	rs402710†	CLPTM1L	5p15	99.6%	C	1.22 (1.13-1.32)
McKay, 2008 [4]	rs1051730	CHRNA 5, CHRNA 3	15q25	98.8%	T	1.35 (1.5-1.45)
You, 2009 [24]	rs4083914	RGS17	6q23-25	99.7%	G	1.80 (1.36-2.39)
Miki, 2010 [23]	rs4488809	TP63	3q28	100%	T	1.27 (1.14-1.41)

*Characteristics of the loci from the cited genome-wide association and fine-mapping studies. CI, confidence interval; OR, odds ratio.

†Rs402710 showed relatively litter LD with rs2736100 (D’ = 0.020).

‡ Odds ratio per copy of the risk allele, as reported in the cited study.

Blood samples were collected from each subject at the time of recruitment, and genomic DNA was extracted using QIAamp DNA Maxi kit (Qiagen GmbH). All SNPs were determined using the Sequenom MassARRAY iPLEX platform using the matrix-assisted laser desorption/ionization time-of-flight mass spectrometer (MALDI-TOF). Primer sequences are available on request. Overall, more than 98% of genotypes were successfully determined for all the SNPs; 5% of samples were randomly selected to re-genotype for quality control, and showed a reproducibility of 100%.

### Genetic risk score computation

Two approaches were used to calculate the genetic risk score (GRS): a simple risk alleles count method (count GRS, cGRS) and a weighted method based on the genotype frequencies for each SNP and effect sizes (allelic odds ratio) from our study (weighted GRS, wGRS). Based on the log-additive model, the three genotypes AA, AB, and BB (A, low-risk allele; B, high-risk allele) for an SNP had a relative risk of 1, OR and OR^2^, respectively. If the B allele had frequency p, then the average relative risk in the population is calculated as: u = (1-p)^2^ + 2p (1-p) OR + p^2^OR^2^. The adjusted risk values for AA, AB, and BB genotype were 1/u, OR/u, and OR^2^/u^2^, respectively. Missing genotypes were assigned a value of 1. The formula for our combined SNP weighted risk score was: wGRS = SNP1 × SNP2 × SNP3 × SNP4, where SNP1-4 were weighted risk score for individual SNPs.

### Percentage of genetic variance explained

The percentage of genetic variance was estimated under a liability threshold model [16]. Allele frequencies and effect sizes corresponding to ORs were used to calculate the threshold: [2p (1-p)] β^2^ (p, risk allele frequency; β, additive allelic effect).

### Statistical analysis

Logistic regression was employed to test the association between genetic variants and lung cancer risk. The classification ability of the model was assessed using the area under the receiver operating characteristic (ROC) curve (AUC), known as a concordance (c) statistic. The Hosmer-Lemeshow test was used to evaluate the calibration of risk estimated in our cohort data. Internal validation of models was carried out using a bootstrap method involving 1000 replications to adjust model parameters for potential over-fitting. A second validation was performed by randomly dividing the cohort population into two unequal groups (one with 75% of the population, and the second with the remaining 25%). The larger group (training set) was used to rebuild the same model, which was then tested on the remaining 25% of the population (test set). All analyses were conducted by Statistical Analysis System (SAS) software (version 8.2; SAS Institute, Cary, NC). All p values were two-sided, and *p* values < 0.05 were considered statistically significant.

## Results

### Association between genetic risk alleles and lung cancer

Five lung cancer-associated SNPs identified in previous GWA studies were evaluated in this study (Table 1). Each SNP was in Hardy-Weinberg equilibrium (*p* > 0.05) in the control group. The results for the selected risk alleles with lung cancer are shown in Table 2. Four SNPs (rs2736100, rs402710, rs4083914, and rs4488809) were significantly associated with lung cancer in our study. Rs2736100 and rs402710 displayed weak linkage disequilibrium (D’ = 0.022), but each was still associated with lung cancer risk after adjusting for the other. The four significantly associated SNPs were selected for further analysis. A liability threshold model was used to estimate the percentage of genetic variation explained by each of the risk alleles. Our data showed that rs2736100, rs402710, rs4083914 and rs4488809 SNPs accounted for 1.33%, 0.40%, 0.47% and 1.82% of the genetic variance, respectively (Table 2).

**Table 2:** Association between SNPs and lung cancer

**SNP**	***P***_**HWE**_	**Frequency of high-risk allele**	Observed OR, (95% CI)*	***P***	**Genetic variance explained†**
Rs2736100	0.49	0.41	1.18 (1.09-1.27)	< 0.001	1.33%
Rs402710	0.43	0.68	1.10 (1.01-1.19)	0.034	0.40%
Rs1051730	0.05	0.02	1.09 (0.85-1.40)	0.108	-
Rs4083914	0.25	0.14	1.15 (1.03-1.28)	0.013	0.47%
Rs4488809	0.50	0.47	1.21 (1.11-1.30)	< 0.001	1.82%

NOTE: CI, confidence interval; OR, odds ratio.

*Odds ratio per copy of the high-risk allele.

†The percentage of genetic variance was estimated under a liability threshold model: [2p (1-p)] β2 (p, risk allele frequency; β, additive allelic effect).

### Genetic risk score association

**Figure 1:**
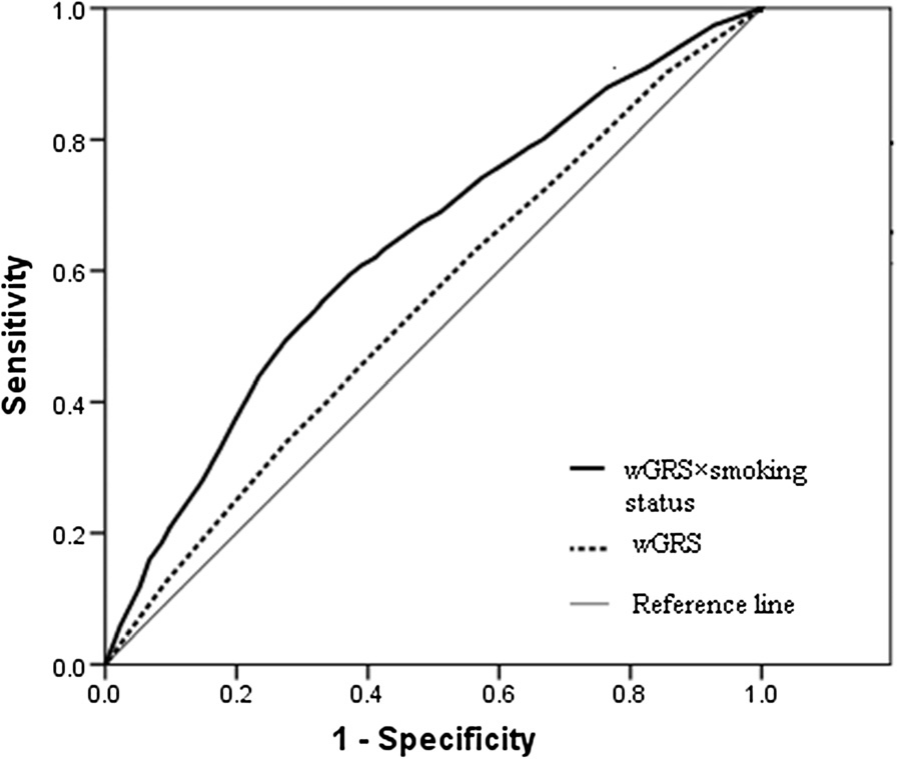
**Receiver operating characteristic curve for wGRS × smoking status and wGRS,** Straight line indicates the null discrimination. wGRS, weighted genetic risk score.

Associations between lung cancer risk and genetic risk score based on two methods (cGRS and wGRS) were evaluated and the results are shown in Table 3. To estimate the risk of genetic risk score, we calculated odds ratios according to wGRS deciles. Compared with participants who were in the lowest decile, those in the highest decile had a 2.01-fold (95% confidence interval (CI), 1.59-2.54; *p* < 0.001) increased risk of lung cancer. We next compared the discriminative ability of GRS by calculating the AUC, and wGRS was shown to be significantly better than cGRS in lung cancer risk prediction (Table 3 and Figure 1). The AUC was 0.551 for wGRS versus 0.542 for cGRS (*p* < 0.001).

**Table 3:** Distribution of cGRS, wGRS, and demographic characteristic of case patients and control subjects

**Variable**	**Case no. (%)**	**Control no. (%)**	**Logistic regression† OR (95% CI)**	***P *for trend**	**ROC AUC/c statistic**
					**(95% CI) (BOC)**
cGRS (count risk allele)					
0-1	125 (5.48)	226 (8.11)	1.00 (ref.)	<0.001	0.542 (0.525-0.557)‡
2	337 (14.76)	465 (15.70)	1.31 (1.01-1.70)		(0.540)*
3	589 (25.80)	733 (26.32)	1.45 (1.14-1.85)		
4	600 (26.28)	740 (26,57)	1.47 (1.15-1.87)		
5	435 (19.05)	453 (16.27)	1.74 (1.35-2.34)		
≧6	197 (8.63)	168 (6.03)	2.12 (1.57-2.86)		
wGRS (weighted genetic risk score)					
0 (<Q10)	222 (9.72)	400 (14.36)	1.00 (ref.)	<0.001	0.551 (0.532-0.564)‖
1 (Q10-Q20)	192 (8.41)	241 (8.65)	1.44 (1.12-1.84)		(0.550)*
2 (Q20-Q30)	211 (9.24)	272 (9.77)	1.40 (1.10-1.78)		
3 (Q30-Q40)	215 (9.42)	299 (10.74)	1.30 (1.02-1.65)		
4 (Q40-Q50)	423 (18.53)	512 (18.38)	1.49 (1.21-1.83)		
5 (Q50-Q60)	102 (4.47)	115 (4.13)	1.60 (1.17-2.19)		
6 (Q60-Q70)	140 (6.13)	173 (6.21)	1.46 (1.11-1.92)		
7 (Q70-Q80)	185 (8.10)	195 (7.00)	1.71 (1.38-2.22)		
8 (Q80-Q90))	309 (13.53)	323 (11.60)	1.72 (1.38-2.16)		
9 (≧Q90))	284 (12.44)	255 (9.16)	2.01 (1.59-2.54)		
Age (mean±SD)	60.09±10.29	60.56±9.58			
Age group					
1 (≦60 year)	1113 (48.75)	1360 (48.53)	1.00 (ref.)	0.954	-
2(>60 year)	1170 (51.25)	1425 (51.17)	1.00 (0.90-1.12)		
Gender					
Male	1683 (73.72)	2039 (73.21)	1.00 (ref.)	0.686	-
Female	600 (26.28)	746 (26.79)	0.97 (0.86-1.11)		
Smoking status					
Never	801 (35.09)	1517 (54.47)	1.00 (ref.)	<0.001	0.619 (0.603-0.634)※
Light-smokers	416 (18.22)	542 (19.46)	1.45 (1.25-1.70)		(0.618)*
Heavy-smokers	1066 (46.69)	726 (26.07)	2.78 (2.45-3.16)		

NOTE: CI, confidence interval; OR, odds ratio.

* Bootstrap optimism corrected estimate of model performance based on 1000 bootstrap resampling.

† Log-additive model.

‡ AUC value of model that only contain cGRS.

‡ AUC value of model that only contain wGS.

※AUC value of model that only contain smoking status.

### Discrimination performance of wGRS × demographic characteristics

General demographic characteristics (including age, gender, smoking status) were analyzed (Table 3), and factor associated with lung cancer risk (smoking history) was further evaluated in the risk assessment model. The AUC value of model that only has smoking was 0.619 (0.603-0.634). To estimate the relative risk of lung cancer for individuals with a given combination of risk factors, we used logistic regression to construct an assessment model including the wGRS and smoking status. As shown in Table 4 and Figure 1, the prediction model expressed as follows: OR = exp (−0.9372 + 0.0610 × wGRS + 0.5142 × smoking status). The AUC of full model was 0. 639 (*p*-value for discrimination difference of AUC values for wGRS <0.001; *p*-value for discrimination difference of AUC values for smoking status = 0.024). To correct for potential over-fitting, we adjusted the model parameters using the bootstrap method [17]. As the adjusted and unadjusted values were almost the same, this indicated that there was little over-fitting of the model.

**Table 4:** Logistic regression model including GRS and smoking status

**Factors**	**Coefficient**	***P*-value**	**OR**	**95% CI**
wGRS	0.0610	<0.001	1.06	1.04-1.08
Smoking status	0.5142	<0.001	1.67	1.57-1.78
Constant	−0.9372	<0.001	-	-
Model performance statistics				
Hosmer-Lemeshow	0.154			
Goodness of fit				
ROC AUC/c statistics (95% CI) (BOC)*	0.639 (0.621-0.652) (0.637)*			

* Bootstrap optimism corrected estimate of model performance based on 1000 bootstrap resampling.

The adjusted AUC for the full model described above was 0.637 (Table 4 and Figure 1). The contribution of wGRS to the model was 0.020 (assessed by the reduction in c statistic when wGRS was removed from the full model). Smoking status was the strongest predictor in the model, with a contribution of 0.088.

### Internal model validation

After the model was rebuilt on the training set, it displayed similar discrimination ability to the original one (c statistic, 0.641). The model was then tested on the test set, and also showed similar discrimination ability (c statistic, 0.633).

We selected a cut-off value corresponding to the maximum sensitivity and specificity. Predictive performances of the rebuilt model for defining a high risk group were then assessed by sensitivity, specificity, accuracy, positive predicted value (PV+) and negative predicted value (PV-) (Table 5). The predictive performances of this model in the two separated groups were similar (accuracy of training set: 61.72%; accuracy of test set: 61.48%).

**Table 5:** Predictive performance of model

**Dataset**	**Accuracy**	**Sensitivity**	**Specificity**	**PV+**	**PV-**
Training set (75%)	61.72%	54.74%	67.53%	58.40%	64.18%
Test set (25%)	61.48%	52.57%	68.44%	56.52%	64.90%
Overall	61.66%	54.23%	67.76%	57.96%	64.36%

NOTE: PV+, Positive Predicted Value (true positive/ total predicted positive); PV-, Negative.

predicted value (true negative/total predicted negative).

## Discussion

In this study, we systematically evaluated the clinical utility of five SNPs identified in recent GWAs and large cohort studies of lung cancer. Using data from a large case–control study that enrolled 5,068 participants, we found that most of the genetic variants (rs2736100, rs402710, rs4488809, and rs4083914) identified previously in other populations were also associated with risk of lung cancer in a Chinese population. In addition, we showed that a wGRS accounting for the adjusted effect size of each SNP was a better predictor than a cGRS, and had a stronger association with lung cancer risk than any single SNP alone. Although the weighted genetic risk score had a moderate predictive ability, it gave a better discrimination between lung cancer cases and cancer-free controls (AUC of ROC curve, 0.639) when used in combination with smoking status using the logistic regression model.

Several lung cancer risk assessment models have previously been proposed [12-15], but most predictors focused on traditional risk factors such as family history of lung cancer, smoking status, environmental exposure, age and gender. In contrast to these, genetic scores derived from inherited genetic variations offer the advantage of stability during the lifetime of the individual.

Previous studies have indicated that inherited genetic variants might account for an important fraction of lung cancer developmental risk [18,19]. Recent GWA studies of lung cancer in population of European ancestry identified three lung cancer susceptibility loci: 5p15 (TERT-CLPM1L), 15q25 (CHRNA 3–5) and 6p21 (BAT3-MSH5) [4-9]. McKay et al. [4] reported two independent markers of lung cancer at the 5p15 region, rs2736100 (TERT) and rs402710 (CLPM1L). Furthermore, an association between rs2736100 and lung cancer were also replicated in Asian populations [20,21]. Of the five SNPs evaluated in this study, we observed a strong signal at rs2736100 in accordance with previous reports.

15q25 region encoding nicotinic acetylcholine receptor subunits was thought to be related with lung cancer risk [6-8]. We evaluated the rs1051730 SNP from this region in the present study, but it showed no association with disease risk. It is conceivable that the rs1051730 allele frequency in the Chinese Han population (MAF, 0.02) is too low to confirm the effects seen in European populations [22]. Reported risk SNPs at 6p21 (rs3117582 and rs3131379) are not polymorphic in the Chinese Han population, so were excluded from this study. Rs4488809 and rs4083914, previously identified by GWA and large cohort investigations, were also shown to be significantly associated with lung cancer risk in this study [23,24].

Of the five SNPs evaluated in this study, the strongest signal was found for rs4488809, for which there was 21% elevated risk of lung cancer with each risk allele. The three other SNPs (rs2736100, rs402710, and rs4083914) were also associated with a risk of lung cancer, albeit at lower levels (<18%) for each risk allele. The estimated proportion of genetic variation explained by these four SNPs was therefore 4.02%, which includes 1.82% due to rs4488809 and 1.33% due to rs2736100. This suggests that the genetic susceptibility loci identified by GWA and large cohort studies in other populations only confer a small to moderate risk in a Chinese population when considered alone, and are of little use in lung cancer risk assessment.

To overcome this, a genetic risk score combining multiple loci might improve the identification of persons at high risk for developing lung cancer. Our results showed that although wGRS was highly associated with lung cancer susceptibility, a model including wGRS alone did not provide a better predictive capacity than a model including traditional factors (c statistic for wGRS alone, 0.551). Smoking history was also associated with lung cancer risk in this study, in agreement with previous reports [12,25]. Moreover, wGRS, in combination with smoking status showed a better predictive ability (c statistic, 0.639). Indeed, the c statistic decreased by 0.020 when wGRS was removed from the full model, indicating that genetic risk factors could improve the discriminatory ability of the traditional assessment model, although this effect was moderate.

This study has a number of limitations. First, the susceptibility loci identified by GWA and large cohort studies with evidence of replication were associated with a lung cancer risk through strong linkage disequilibrium, and always conferred moderate effects. Many additional susceptibility loci for lung cancer remain to be discovered, and it is possible that rare variants with high penetrance would explain the remaining hereditary [26]. Next generation sequencing technologies offer hope in the future research of such variants [27]. Recently, several identified SNPs were reported [28-30]. Combining these new SNPs might result in improvement in classification of lung cancer risk. Second, because of limited traditional factors, the full predictive model established in this study only provided a moderate level of classification accuracy, with a c statistic of 0.639, which is inadequate for risk prediction. The discriminatory capability of our model might be improved by including additional factors such as history of bronchitis, emphysema or pneumonia, asbestos exposure, and family history of lung cancer. Third, our assessment model lacked external validation even though our estimates of ROC AUC were corrected for over-fitting by bootstrap and internal validation was conducted. Finally, as this was a retrospectively designed study, the results need to be validated by a large-scale, prospective study.

## Conclusions

We have shown that most of the genetic susceptibility loci identified by previous GWA and large cohort studies in other populations were also associated with lung cancer risk in a Chinese population. Although the weighted genetic risk score had only a moderate discriminatory accuracy, it still improved the predictive ability of the assessment model, which might help in the identification of individuals at a high risk of developing lung cancer. Future studies should focus on establishing a risk assessment model that incorporates both genetic variants and established traditional factors for lung cancer.

## Abbreviations

95% CI: 95% confidence interval; AUC: Area under the curve; BOC: Bootstrap optimism corrected; GRS: Genetic risk score; GWAS: Genome-wide association (GWA) studies; OR: Odds ratio; ROC: Receiver operating characteristic; SNP: Single nucleotide polymorphism.

## Competing interests

The authors declare that they have no competing interests.

## Authors’ contributions

HL, LXY, DRL, WMW and LJ conceived and designed the experiments. HL, LXY and XYZ performed the experiments. HL and XYZ analyzed the data. JQ, JCW, HYC, WWF, HCL and LJ contributed reagents, materials or analysis tools. DRL, WMW and HL wrote the manuscript. All authors read and approved the final manuscript.

## Pre-publication history

The pre-publication history for this paper can be accessed here:

http://www.biomedcentral.com/1471-2350/13/118/prepub
